# Prevalence and Associated Factors of Depressive Symptoms among Chinese Underground Coal Miners

**DOI:** 10.1155/2014/987305

**Published:** 2014-02-23

**Authors:** Li Liu, Lie Wang, Jie Chen

**Affiliations:** ^1^Department of Social Medicine, School of Public Health, China Medical University, 92 North 2nd Road, Heping District, Shenyang, Liaoning 110001, China; ^2^Department of Occupational and Environmental Health, School of Public Health, China Medical University, 92 North 2nd Road, Heping District, Shenyang, Liaoning 110001, China

## Abstract

Although underground coal miners are quite susceptible to depressive symptoms due to a highly risky and stressful working environment, few studies have focused on this issue. The purpose of the study was to evaluate the prevalence of depressive symptoms and to explore its associated factors in this population. A cross-sectional survey was conducted in a coal-mining population in northeast China. A set of self-administered questionnaires was distributed to 2500 underground coal miners (1,936 effective respondents). Depressive symptoms, effort-reward imbalance (ERI), overcommitment (OC), perceived physical environment (PPE), work-family conflict (WFC), and some demographic and working characteristics were measured anonymously. The prevalence of depressive symptoms was 62.8%, and the mean level was 20.00 (9.99). Hierarchical linear regression showed that marital status, education, monthly income, and weekly working time were significantly associated with depressive symptoms. A high level of depressive symptoms was significantly associated with high ERI, PPE, WFC, and OC. Accordingly, most Chinese underground coal miners probably have depressive symptoms that are mainly predicted by some occupational psychosocial factors. Efforts should be made to develop strategies to reduce ERI and OC, improve physical working environment, and care for workers' family well-being, thereby mitigating the risk of depression among Chinese underground coal miners.

## 1. Introduction

Depression is a common mental health problem in the workplace worldwide. Previous studies on diagnostic assessment of depression in the working population indicated high prevalence across occupations and countries [1, 2]. In Canada, 4.6% of the employed population aged from 15 to 75 years experienced an episode of depression in the previous year according to the Community Health Survey [1]. Even higher estimated prevalence of 12-month major depressive disorder (6.4%) has been reported among American workers [2]. Moreover, higher prevalence of depressive symptoms in workplaces has been examined, with studies from different countries reporting the prevalence of at least 20% [3–6]. In particular, with the rapid development and reform of China's society and economy, many Chinese occupational groups are facing a variety of mental health problems, especially depression. The average prevalence of depressive symptoms was 46.2% among various occupational groups in China, mainly including teachers, community health workers, traffic police, researchers, foreign enterprise employees, and managers [6].

Besides its high prevalence, the adverse effects of depression are extensive in the workplace. Depression, the leading cause of disability worldwide, can affect workers' productivity and overall job performance [7]. Depressed workers have high rates of absenteeism [8], presenteeism [8], and turnover intention [9], even work cessation, and they are more likely to abuse alcohol and drugs in comparison with those without depression [10]. Moreover, the risk of occupational injury experience was higher among workers who reported depressive symptoms [11]. If not recognized or treated, depression can profoundly impair workers' quality of life [12].

In view of its high prevalence and extensive adverse effects, the associated factors and pathogenesis of depression should be recognized as global research priorities in order to develop effective strategies for depression prevention and treatment in workplaces. Demographics, working characteristics, and perceived occupational psychosocial factors (such as job stress, interpersonal relationship, work-life interference, perceived social support, and threat perception) have been identified as predictors of depression or depressive symptoms [13]. While many factors are common across different occupations, some special occupational groups could present some unique issues. These differences could create substantial obstacles to developing a universal depression prevention and treatment strategy in the workplace. Therefore, research on associated factors and pathogenesis of depression in different occupational groups will help develop population-specific and effective intervention.

Underground coal miners, as a special occupational group, perform a number of tasks in a confined space, such as weightlifting, bending, and prolonged standing. Many different types of physical agents coexist in their underground working environment, including noise, vibration, temperature, and humidity. The complex working conditions of underground miners seriously threaten their safety and health. Moreover, poor production organization and various occupational psychosocial factors are prone to adversely affect the whole health of underground miners [14]. Current studies in this population mainly focused on the adverse effects of their specific working environment on physical health [15–17]. However, very limited studies paid attention to mental health in mining industry workers though it is an important public health issue, especially in relation to the associations between occupational psychosocial factors and mental health problems. Several previous studies indicated that miners suffered various mental health problems, especially depression, anxiety, and substance abuse [18–21]. McLean's study confirmed that relationships, life styles, work characteristics, and mental health attitudes were the main factors that could impact the mental health and psychological well-being of resident (non-fly-in fly-out) mine workers at a local mine in regional Queensland [22]. Even several studies on mining community samples reported their poor mental health [23–25]. To our knowledge, it is still unknown whether there is a high prevalence of depressive symptoms among underground coal miners. If so, it may be prone to result in various adverse effects in the underground working environment, even increase the probability of safety accident.

Especially in China, as the main body of national primary energy production and consumption, coal resource accounts for about 70% in recent years. At present, there are about six million underground coal miners in China [26]. Most of them often have to face high job demands, low social status, workplace discrimination, strict safety regulations, irregular life, and work-life interference, which are likely to negatively impact their mental health [27, 28]. In particular, the mental health problems of underground miners are more severe than those of surface workers who suffered from at the same coal mine [21]. Although working conditions and organizational arrangement of underground coal miners have been optimized and improved according to the Law of the PRC on Safety in Mines, the impacts of various occupational psychosocial factors on mental health will continue for a long period of time. Therefore, in-depth study on prevalence and associated factors of depression is urgently needed to provide scientific basis for depression prevention and treatment targeted at underground coal miners.

In light of the above concerns, the purpose of this study was to assess the prevalence of depressive symptoms and investigate their associated factors among Chinese underground coal miners. We hypothesized that the depressive symptoms of underground coal miners might be highly prevalent. With consideration on previous studies and Chinese-specific culture, demographics (age, marital status, education, and chronic disease), working characteristics (job rank, monthly income and weekly work time), and some occupational psychosocial factors including effort-reward imbalance (ERI), overcommitment (OC), perceived physical environment (PPE), and work-family conflict (WFC) were investigated to clarify the associated factors of depressive symptoms in this population.

## 2. Materials and Methods

### 2.1. Study Design and Sample

A cross-sectional survey was conducted in a coal-mining population in northeast China and data were collected from July to August 2013. Twenty-five hundred underground miners were cluster-sampled from six coal mines. About 50% of underground miners from each department of selected coal mines were sampled. Notably, all underground miners were male. After the participants were given a brief and complete description of the study, written informed consent was obtained. The participants completed a set of self-administered questionnaires anonymously after their scheduled shifts with the help of trained investigators. There was no interference caused by investigators in the process of completing questionnaires. Missing data concerning any item within questionnaires was excluded from the final analysis. Complete responses were obtained from 1,936 individuals (response rate: 77.4%). The study was approved by the Committee on Human Experimentation of China Medical University, and the study procedures were in accordance with ethical standards.

### 2.2. Measures

The Chinese version of the Center for Epidemiologic Studies Depression (CES-D) Scale was used to measure depressive symptoms [29, 30]. The scale consists of 20 items with 4 options that describe the frequency of respondents' feelings in the past week ranging from 0 “rarely or none of the time (less than 1 day)” to 3 “most or all of the time (5 to 7 days).” The severity of depressive symptoms increases with a higher summed score that ranges from 0 to 60. To estimate the prevalence of depressive symptoms, a CES-D score of ≥16 was defined as “depressive symptoms” in this study [4, 6, 31]. The CES-D scale has demonstrated good reliability and validity in the Chinese occupational population [6, 31]. In the present study, Cronbach's alpha coefficient for the CES-D scale was 0.89.

The Chinese version of the Effort-Reward Imbalance scale was used to assess the levels of ERI and OC [32, 33], and it has been widely used among Chinese occupational populations with good reliability and validity [33, 34]. The ERI scale consists of three subscales: effort (6 items), reward (11 items), and OC (6 items). For the effort and reward subscales, the participants initially express their attitude towards a work situation (“agree” or “disagree”) and then evaluate what extent (from “not distressed” to “very distressed”) they usually feel distressed. For the OC subscale, responses are scored from 1 “strongly disagree” to 4 “strongly agree.” From the ERI scale, extrinsic ERI and intrinsic OC are two independent measures of occupational stress. The ERI was calculated according to the predefined formula ERI = effort/(reward ∗ 0.5454). Higher ERI indicates higher level of imbalance. Those who score high on OC expend inordinate amounts of intrinsic effort that is not met by the externally defined rewards received in turn. In this study, the Cronbach's alpha for the effort, reward, and OC subscales was 0.86, 0.92, and 0.71, respectively.

PPE was assessed using a 10-item physical environment subscale derived from the Chinese version of the Occupational Stress Inventory-Revised Edition (OSI-R) [35, 36]. Each item has five responses with categories ranging from 1 “never” to 5 “very often.” Total score for the physical environment scale was calculated as indicator of PPE in this study, with higher PPE score indicating harsher physical environment in workplaces. Adequate reliability and validity of the subscale of the OSI-R have been demonstrated across multiple Chinese samples [36, 37]. For the physical environment scale, Cronbach's alpha was 0.79 in this study.

WFC was measured by the 9-item Work-Family Conflict Scale (WFCS) [38, 39]. It measures the extent to which work demands interfere with family-related obligations [38]. Each item has five responses with categories ranging from 1 “strongly disagree” to 5 “strongly agree.” Responses were averaged to get an average score for WFC. Higher value indicated higher level of WFC. The Chinese version of the WFCS has demonstrated good reliability and validity [39, 40]. In the present study, the Cronbach's alpha for the WFCS was 0.91.

Demographics collected from each respondent included age, marital status, education, and chronic disease in this study. Marital status was categorized as single/divorced/widowed/separated or married/cohabiting. Education was categorized as junior high school or under, senior high school/technical secondary school, and junior college or above. Chronic disease was defined as “yes” if the respondents had ever been diagnosed with any common chronic disease (e.g., hypertension, hyperlipidemia, gastritis, arthritis, hepatic steatosis, and diabetes).

Working characteristics were assessed on the basis of three items: job rank, monthly income (RMB, yuan), and weekly working time (hours) in this study. Job rank was categorized as head miner and staff miner. Monthly income was categorized as ≤3000 yuan, 3001–5000 yuan, and >5000 yuan. Weekly working time was collected as a continuous variable and was also divided into ≤40 hours or >40 hours groups according to the current work arrangement of 8 hours per day in China.

### 2.3. Statistical Analysis

Before the data analyses were conducted, the normal distribution of continuous variables was verified using the P-P-plot and *K*-*S* tests. Comparisons of depressive symptoms for demographic and working characteristics were examined by Student's *t*-test or one-way ANOVA. Pearson's correlation analyses were executed to examine the correlations of continuous independent variables with depressive symptoms. Hierarchical linear regression analysis was performed to examine the associations between independent variables and depressive symptoms. In Block 1, demographics (age, marital status, education, and chronic disease) were put in the model. Because marital status, education, and chronic disease are categorical variables, dummy variables were set for these three variables, respectively. For marital status, “single/divorced/widowed/separated” was set as the reference group. For educational level, “junior high school or under” was set as the reference group. For chronic disease, “yes” was set as the reference group. Working characteristics (job rank, monthly income, and weekly work time) were added in Block 2. Because job rank and monthly income are categorical variables, dummy variables were set for these two variables, respectively. For job rank, “head miner” was set as the reference group. For monthly income, “≤3000 yuan” was set as the reference group. In Block 3, ERI, OC, PPE, and WFC were added. Variances of depressive symptoms explained by different groups of independent variables were examined by Δ*R* ^2^. Moreover, tolerance and variance inflation factor (VIF) were used to check for multicollinearity. All analyses were conducted using SPSS for Windows, Ver. 13.0.; Statistical significance was defined as *P* < 0.05 (two-tailed).

## 3. Results

### 3.1. Demographic and Working Characteristics of Subjects

Demographic and working characteristics of study subjects are presented in Table 1. The prevalence of depressive symptoms among Chinese underground coal miners was 62.8%. Nearly half (48.7%) of the respondents were in the age group of 41–50 years and 83.0% were married/cohabiting. Among all respondents, 49.8% had received at least senior high school/technical secondary school education, and 36.7% had been diagnosed with at least one chronic disease. For working characteristics, 79.6% of respondents were head miners, and 58.3% had monthly income between 3001 and 5000 yuan RMB and 73.2% worked > 40 hours per week.

### 3.2. Differences of Depressive Symptoms in relation to Demographic and Working Characteristics

Univariate analyses of depressive symptoms in relation to demographic and working characteristics are shown in Table 2. The mean score of depressive symptoms was 20.00 (9.99). There was no significant association of depressive symptoms based on age, chronic disease, job rank, and weekly working time. Marital status, education, and monthly income were significantly related to depressive symptoms. For marital status, single/divorced/widowed/separated respondents reported significantly higher depressive symptoms than those married/cohabiting (*t* = 2.68, *P* = 0.007). The post hoc SNK test indicated that the level of depressive symptoms was significantly higher among respondents with junior high school or under education compared with those with junior college or above education; respondents earned ≤ 3000 yuan and 3001–5000 yuan groups reported significantly higher depressive symptoms than those earned > 5000 yuan group, respectively.

### 3.3. Correlations of Continuous Independent Variables with Depressive Symptoms

Correlations of continuous independent variables with depressive symptoms are detailed in Table 3. The average age of respondents was 40.18 (9.25) years. Age was not correlated with depressive symptoms. Weekly working time, ERI, OC, PPE, and WFC had positive correlations with depressive symptoms in this study.

### 3.4. Factors Associated with Depressive Symptoms

Hierarchical linear regression analyses of the factors associated with depressive symptoms are presented in Table 4. In Block 1, age, marital status, education, and chronic disease were significantly associated with depressive symptoms. When working characteristics were added in Block 2, age, education, monthly income, and weekly working time were significantly associated with depressive symptoms. In Block 3, among these demographic and working characteristics, marital status, education, monthly income, and weekly working time were significantly associated with depressive symptoms. In addition, a high level of depressive symptoms was significantly associated with high ERI, PPE, WFC, and OC (in descending order of standardized estimate). Results of hierarchical linear regression analyses also showed that the groups of demographics, working characteristics, and occupational psychosocial factors explained 1.3%, 1.2%, and 14.7% of variance in depressive symptoms, respectively.

## 4. Discussion

This study assessed the prevalence of depressive symptoms and explored the factors associated with depressive symptoms among Chinese underground coal miners. Findings show that most Chinese underground coal miners are seriously suffering from depressive symptoms, and some demographics, working characteristics, and occupational psychosocial factors are associated with depressive symptoms. These results could help coal mine administrators increase their understanding of depressive symptoms and develop effective interventions for prevention and treatment targeted at underground coal miners.

The mean level of depressive symptoms was 20.00 (9.99) for Chinese underground coal miners in this study. Compared with other male occupational groups, this level was higher than 17.13 (8.85) for that of various occupations from Shanghai [6] and 17.70 (10.41) for non-frontline correctional officers [31] in China; it was consistent with 19.10 (11.16) and 19.54 (10.54) derived from previous studies on Chinese frontline correctional officers [31] and doctors [34], respectively. Also, it was higher than that of internal migrant workers with a score of 10.4 (8.6) [41] and a community sample with a level of 18.3 (9.3) [42] in China. In addition, the prevalence of depressive symptoms was 62.8% measured by a CES-D score of 16 or more in our underground miners, which was higher than that of other male occupational groups including various occupations 47.2% from Shanghai [6], frontline correctional officers 59.7%, and nonfrontline correctional officers 56.2% [31] in China but was lower than that of male Chinese doctors 66.6% [43]. Without regard to gender, the prevalence of depressive symptoms was higher than that of various occupations 46.2% from Shanghai [6] and internal migrant workers 23.7% [41] and was consistent with Chinese female nurses 61.7% [44]. The prevalence was also higher than that of a mining community sample in Italy [24, 25]. However, it is notable that other instruments are used to measure depressive symptoms in the Italian mining community sample mentioned above.

For the CES-D scale, a cut-off score of 16 is determined originally to identify potential clinical depression [29]. Previous studies regarding Chinese populations suggest that the original cut-off score of 16 has low positive predictive value (PPV) and may be too low for the Chinese people [45, 46]. Meanwhile, it should be recognized that there is the possibility that a choice of 16 as cut-off score may have inflated the number of false positives. Obviously, more in-depth research is needed to determine a new and generally accepted cut-off score of the CES-D scale among different Chinese populations. Given the complexity of the cutoff score selection, we applied the previous study methodology that defined three groups of depressive symptoms: mild (total score = 16–20), moderate (total score = 21–26), and severe (total score > 26) [42, 47, 48]. Based on the CES-D scale scores, 340 respondents (17.6%) had mild depressive symptoms, 379 (19.6%) had moderate depressive symptoms, and 497 (25.7%) had severe depressive symptoms among underground coal miners.

Therefore, our results indicated that most Chinese underground coal miners suffered seriously from depressive symptoms. This finding would promote coal mine administrators to be concerned with the risk of depressive symptoms. Efforts should be done to prevent and reduce depressive symptoms among Chinese underground miners.

Among demographic characteristics, marital status and education were related to depressive symptoms. Since supportive functions from spouse might protect mental health, depressive symptoms were expected to be more severe among single/divorced/widowed/separated miners in comparison with those married/cohabiting miners in the same working environment [31, 49]. Obviously, the spouses partially share family responsibility and financial burden in daily life [49]. As an occupational population with relatively low level of education, the whole health of underground coal miners might be more sensitive to the positive effects of education in comparison with those occupational groups with relatively higher educational level. Higher education could be a protective factor for the mental health of underground coal miners [50].

Among working characteristics, monthly income and weekly working time were associated with depressive symptoms in the final results of this study. Unsurprisingly, for many underground miners, the main motivation for mining was the money afforded. Because it is very difficult to do anything else once workers begin earning a mining wage, income level is directly linked to their social status and quality of life. For the same position, the salary varies within a considerable range due to performance-based management measures in China, which suggests that the miners with a higher monthly income might be satisfied with more sense of achievement, which is one approach to reducing depressive symptoms in workplaces [51]. Long working time is an important occupational stressor that could result in mental health problems among various occupational groups [13, 51, 52]. Underground miners could not get sufficient rest due to more working time. Moreover, little time spent on family and interpersonal communication could increase their work-life interferences and psychological strains. In this study, participants worked > 40 hours per week reported higher WFC compared with those who worked ≤ 40 hours per week.

The associations of ERI, PPE, WFC, and OC with depressive symptoms were also found among Chinese underground coal miners [5, 6, 34, 44, 52]. ERI had stronger association with depressive symptoms than the other occupational psychosocial factors. Due to the extremely harsh environment, underground miners not only often face dangerous situations, but also have to work all hours of day and night, weekends, and holidays on rotating shift. However, they do not have high social status and good job prospects, which may cause emotional distress and interpersonal conflict and thereby deteriorate mental health. Overall, the high effort of miners is not met with adequate reward. As a result, underground coal miners in failed social reciprocity could have high level and prevalence of depressive symptoms.

PPE was found to be slightly associated with depressive symptoms in this study, which indicated that the specific working environment adversely affects underground coal miners' mental health. Although the safety of miners substantially increases in the underground working environment, because many health risk factors are well controlled through the implementation of strict control measures, the impact of PPE on mental health still exists.

The role expectations of work and family are always incompatible for many occupational groups. WFC refers to the increasingly common spillover of occupational roles and duties into family life, whereas family-work conflict (FWC) results when family responsibilities impair workers' job performance [38]. In comparison with other occupational populations, underground coal miners are devoting much time and energy to work, because they need to adhere to a strict roster, and experiencing higher level of WFC in China. Thus, the potential impact of WFC on depressive symptoms was concerned in this study. In correspondence with the results of previous studies [5, 53], WFC was found to be positively related to depressive symptoms.

In addition, OC was also found to be associated with depressive symptoms among underground coal miners. This result indicated that OC may contribute to the development of depressive symptoms [6, 34, 44, 52]. As a personal cognitive pattern of coping with work, a high level of OC increases the risk of reduced health in the working population. Overcommitted individuals are proposed to work harder than those demanded for a given task. Thus, OC can lead to job burnout, especially emotional exhaustion, which has been found to overlap with depression.

Since it is impossible to avoid the specific working environment for underground coal miners, programs focused on raising awareness and promoting prevention; early identification and effective treatment of depressive symptoms can improve both health and workplace outcomes and help miners thrive at work. According to the results of our study, some effective strategies should be implemented with Chinese underground coal miners to relieve depressive symptoms. On one hand, many sources of work-related stress can be reduced or potentially eliminated with proactive workplace policies. Coal mine managers should encourage a balance between work demands and rewards of underground coal miners. Underground coal miners should avoid OC through developing effective measures for coping with work demands. On the other hand, coal mine managers need to further maintain the strict management and optimization on physical environment and make workers far away from the physical agents' threats. Reasonable working time arrangement not only decreases the workload of underground coal miners, but also enables them to have more time to devote to family life. The Chinese government is vigorously promoting the four six-hour shifts schedule to minimize underground working time. At the same time, coal mine managers also need to pay attention to employees' family well-being. Thus, WFC would be eased for underground coal miners.

However, several limitations of this study must be acknowledged. Firstly, the cross-sectional design of our study may make it impossible to draw causal relations among study variables. The results need to be confirmed in prospective settings. Secondly, the study population comprised only underground coal miners from a coal-mining population in northeast China. However, our study had a large sample size and a high effective response rate, which seemed to provide a good representation of underground coal miners and contribute the generalization of our study's findings. Thirdly, this study was a preliminary investigation and as such did not address a wide range of factors related to depressive symptoms among occupational groups (e.g., stress sensitivity and cognition, supportive organizational climate, psychological capital, and coping style). Finally, the unique use of self-report measures to detect the study variables may affect the correlations between the measures. Some effective quality control measures have been carried out in the process of completing questionnaires in order to minimize this potential problem [54].

## 5. Conclusions

Most Chinese underground coal miners probably have depressive symptoms. Depressive symptoms were predicted by such factors as marital status, education, monthly income, weekly working time, ERI, PPE, WFC, and OC. Occupational psychosocial factors were found to be the most robust indicators of depressive symptoms in this population. Our findings underscored the need for coal mine managers to be aware of the risk of depression. Efforts should be made to develop strategies to reduce the levels of ERI and OC, improve physical working environment, and care for workers' family well-being, thereby mitigating the risk of depression among Chinese underground coal miners.

## Figures and Tables

**Table 1:** Demographic and working characteristics of study subjects.

Variable	*n* (%)
Depressive symptoms	
Yes (CES-D ≥ 16)	1216 (62.8%)
No (CES-D < 16)	720 (37.2%)
Age (years)	
≤30	382 (19.7%)
31–40	417 (21.5%)
41–50	942 (48.7%)
>50	195 (10.1%)
Marital status	
Single/divorced/widowed/separated	329 (17.0%)
Married/cohabiting	1607 (83.0%)
Education	
Junior high school or under	972 (50.2%)
Senior high school/technical secondary school	711 (36.7%)
Junior college or above	253 (13.1%)
Chronic disease	
Yes	711 (36.7%)
No	1225 (63.3%)
Job rank	
Head miner	394 (20.4%)
Staff miner	1542 (79.6%)
Monthly income (RMB, yuan)	
≤3000	451 (23.3%)
3001–5000	1129 (58.3%)
>5000	356 (18.4%)
Weekly working time (hours)	
≤40	519 (26.8%)
>40	1417 (73.2%)

CES-D: the Center for Epidemiologic Studies Depression Scale.

**Table 2:** Univariate analyses of depressive symptoms in relation to demographic and working characteristics.

Variable	Mean ± SD	
Depressive symptoms	20.00 ± 9.99	
Age (years)		*P* = 0.057
≤30	21.25 ± 10.07	
31–40	19.55 ± 9.85	
41–50	19.74 ± 10.01	
>50	19.85 ± 9.89	
Marital status		*P* = 0.007
Single/divorced/widowed/separated	21.35 ± 10.03	
Married/cohabiting	19.73 ± 9.86	
Education		*P* = 0.043
Junior high school or under	20.42 ± 9.82^a^	
Senior high school/technical secondary school	19.91 ± 10.07	
Junior college or above	18.67 ± 10.30	
Chronic disease		*P* = 0.093
Yes	20.51 ± 10.07	
No	19.72 ± 9.93	
Job rank		*P* = 0.216
Head miner	19.45 ± 10.44	
Staff miner	20.15 ± 9.87	
Monthly income (RMB, yuan)		*P* = 0.012
≤3000	20.23 ± 10.09^b^	
3001–5000	20.37 ± 9.88^c^	
>5000	18.59 ± 10.10	
Weekly working time (hours)		*P* = 0.073
≤40	19.34 ± 10.11	
>40	20.25 ± 9.93	

^a^Comparison between subjects with junior high school or under education and those with junior college or above education.

^b^Comparison between subjects with monthly income ≤3000 yuan RMB and those with monthly income >5000 yuan RMB.

^c^Comparison between subjects with monthly income 3001–5000 yuan RMB and those with monthly income >5000 yuan RMB.

**Table 3:** Correlations of continuous independent variables with depressive symptoms.

Variables	Mean ± SD	Correlation with depressive symptoms
Age	40.18 ± 9.25	−0.031
Weekly working time	50.97 ± 10.42	0.083**
ERI^†^	0.69 ± 0.24	0.368**
OC	15.29 ± 2.85	0.179**
PPE	30.51 ± 7.08	0.209**
WFC	3.37 ± 0.77	0.188**

ERI: effort-reward imbalance; OC: overcommitment; PPE: perceived physical environment; WFC: work-family conflict.

^†^The mean of the ERI is logarithmic.

**P < 0.01.

**Table 4:** Hierarchical linear regression analyses of the factors associated with depressive symptoms.

Variables	Block 1 (*β*)	Block 2 (*β*)	Block 3 (*β*)
Age	−0.068*	−0.063*	−0.040
Marital status	−0.055*	−0.049	−0.073**
Education			
Education 1	−0.067*	−0.069*	−0.058*
Education 2	−0.094**	−0.100**	−0.082**
Chronic disease	−0.050*	−0.045	−0.017
Job rank		−0.005	−0.008
Monthly income			
Monthly income 1		−0.007	−0.059*
Monthly income 2		−0.068*	−0.084**
Weekly working time		0.103**	0.047*
ERI^†^			0.298**
OC			0.065**
PPE			0.087**
WFC			0.077**
*F*	5.02**	5.50**	30.65**
*R* ^2^	0.013	0.025	0.172
Δ*R* ^2^	0.013**	0.012**	0.147**

ERI: effort-reward imbalance; OC: overcommitment; PPE: perceived physical environment; WFC: work-family conflict.

^†^ERI is log transformed.

Marital status: married/cohabiting versus single/divorced/widowed/separated; Education 1: senior high school/technical secondary school versus junior high school or under; Education 2: junior college or above versus junior high school or under; Chronic disease: “No” versus “Yes”; Job rank: staff miner versus lead miner; Monthly income 1: 3001–5000 yuan RMB versus ≤3000 yuan RMB; Monthly income 2: >5000 yuan RMB versus ≤3000 yuan RMB.

*P < 0.05, **P < 0.01.
